# Biological Activities of Lactose-Based Prebiotics and Symbiosis with Probiotics on Controlling Osteoporosis, Blood-Lipid and Glucose Levels

**DOI:** 10.3390/medicina54060098

**Published:** 2018-12-03

**Authors:** Arijit Nath, Máté András Molnár, Attila Csighy, Kornélia Kőszegi, Ildikó Galambos, Klára Pásztorné Huszár, András Koris, Gyula Vatai

**Affiliations:** 1 Department of Food Engineering, Faculty of Food Science, Szent István University, Ménesi st 44, HU-1118 Budapest, Hungary; Molnar.Mate@etk.szie.hu (M.A.M.); Attila.Csighy@phd.uni-szie.hu (A.C.); koszegi.laszlone@etk.szie.hu (K.K.); Koris.Andras@etk.szie.hu (A.K.); 2 Soós Ernő Water Technology Research Centre, Faculty of Engineering, University of Pannonia, Üllő út., H-3 Nagykanizsa, Hungary; galambos.i@sooswrc.hu; 3 Department of Refrigeration and Livestock Product Technology, Faculty of Food Science, Szent István University, Ménesi st 43–45, HU-1118 Budapest, Hungary; Pasztorne.Huszar.Klara@etk.szie.hu

**Keywords:** lactose-based prebiotics, probiotics, osteoporosis, blood lipid level, blood glucose level

## Abstract

Lactose-based prebiotics are synthesized by enzymatic- or microbial- biotransformation of lactose and have unique functional values. In this comprehensive review article, the biochemical mechanisms of controlling osteoporosis, blood-lipid, and glucose levels by lactose-based prebiotics and symbiosis with probiotics are reported along with the results of clinical investigations. Interaction between lactose-based prebiotics and probiotics reduces osteoporosis by (a) transforming insoluble inorganic salts to soluble and increasing their absorption to gut wall; (b) maintaining and protecting mineral absorption surface in the intestine; (c) increasing the expression of calcium-binding proteins in the gut wall; (d) remodeling osteoclasts and osteoblasts formation; (e) releasing bone modulating factors; and (f) degrading mineral complexing phytic acid. Lactose-based prebiotics with probiotics control lipid level in the bloodstream and tissue by (a) suppressing the expressions of lipogenic- genes and enzymes; (b) oxidizing fatty acids in muscle, liver, and adipose tissue; (c) binding cholesterol with cell membrane of probiotics and subsequent assimilation by probiotics; (d) enzymatic-transformations of bile acids; and (e) converting cholesterol to coprostanol and its defecation. Symbiosis of lactose-based prebiotics with probiotics affect plasma glucose level by (a) increasing the synthesis of gut hormones plasma peptide-YY, glucagon-like peptide-1 and glucagon-like peptide-2 from entero-endocrine L-cells; (b) altering glucose assimilation and metabolism; (c) suppressing systematic inflammation; (d) reducing oxidative stress; and (e) producing amino acids. Clinical investigations show that lactose-based prebiotic galacto-oligosaccharide improves mineral absorption and reduces hyperlipidemia. Another lactose-based prebiotic, lactulose, improves mineral absorption, and reduces hyperlipidemia and hyperglycemia. It is expected that this review article will be of benefit to food technologists and medical practitioners.

## 1. Introduction

**Figure 1:**
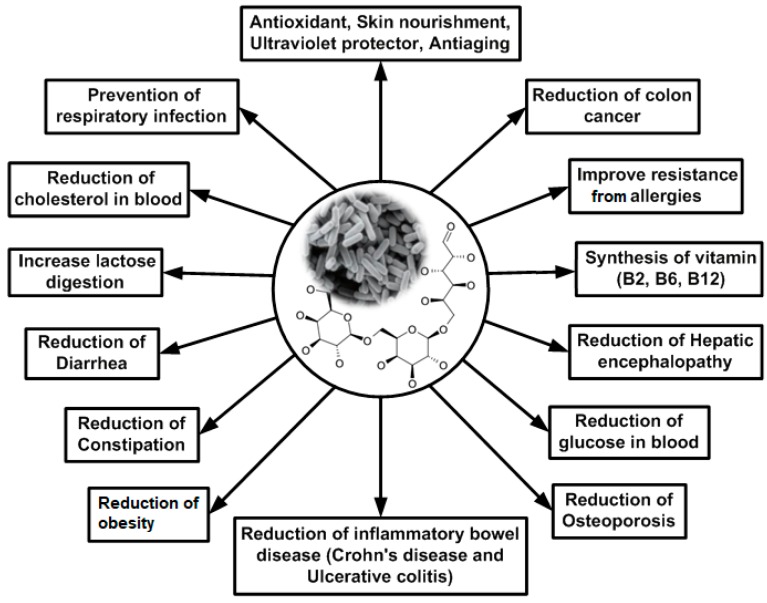
Biological outcomes due to symbiosis of prebiotics and probiotics (self-developed, figure compiled by authors based on Tadesse, 2012 [4]; Al-Sheraji et al., 2013 [5]; Markowiak and Śliżewska, 2017 [6]).

Scientific advancements in biotechnology provide new methods for the synthesis of prebiotics from dairy sources and their applications in food-, biopharmaceutical-, and medical-sectors [1,2]. Prebiotics can be defined as “indigestible fermented food substrates that selectively stimulate the growth, composition and activity of microflora in gastrointestinal tract and thus improve hosts’ health and well-being” [3]. Several biological outcomes due to the interaction of prebiotics with probiotics are presented in Figure 1.

Different types of lactose-derivatives, such as galacto-oligosaccharide, lactosucrose, tagatose, lactulose, lactitol, lactobionic acid, and gluconic acid are produced through different enzymatic reactions (hydrolysis, transgalactosylation, isomerization, fructosyl-transfer, reduction, and oxidation), and microbial fermentation processes. They fulfill all criteria to become a member in the prebiotics family [7,8]. Lactose-based prebiotics are confirmed as ‘safe’ by the Food and Drug Administration Federal agency [9] and have unique biochemical importance [7,8,10]. Because of these reasons, medical practitioners frequently prescribe them as therapeutics. They are recommended for use in a pure form or together with dairy-based products or fruit juices to individuals of all ages [5,11]. When lactose-based prebiotics are consumed alone, their biological activities are expressed via interaction with gut microbiota, already present in intestine [12,13]. Lactose-based prebiotics are resistant from the acid hydrolysis in the stomach, bile salts, and hydrolyzing enzymes in the intestine [10]. However, in the stomach and the upper small intestine of a healthy adult human, microbial population around 10^4^ colony forming units per milliliter, lactose-based prebiotics interact with facultative anaerobic or aerobic microbial communities, including *Lactobacillus* and *Streptococcus* in duodenum (microbial community approximately 10^3^ colony forming units per milliliter); *Lactobacillus*, *Streptococcus*, *Staphylococcus*, and *Veillonella* in jejunum (microbial community approximately 10^4^ colony forming units per milliliter) and *Enterobacteria*, *Enterococcus*, *Bacteroides*, *Clostridia*, *Lactobacillus*, and *Veillonella* in ileum (microbial population approximately 10^6^–10^8^ colony forming units per milliliter), parts of small intestine. Subsequently, they pass to caecum and enhance the activities and survival of the *Bacteroides*, *Clostridia* (*Clostridium coccoides* subgroup and *Clostridium leptum* subgroup). In the large intestine, lactose-based prebiotics interact strictly with anaerobes and obligate anaerobes (microbial population around 10^7^–10^12^ colony forming units per milliliter), such as *Bacteroides*, *Peptostreptococcus*, *Eubacteria*, *Lactobacillus*, and *Clostridia.* Furthermore, in the recto-sigmoidal colon, they interact with *Streptococcus*, *Lactobacillus*, Bifidobacteria, *Bacteroides*, *Clostridia*, and Gammaproteobacteria [14,15]. Lactose-based prebiotics are converted to short chain fatty acids (acetic acid, propionic acid, and butyric acid), lactic acid, and gases (carbon dioxide, methane, and hydrogen) in the presence of gut microbiota. Research has proven that in the proximal colon and distal colon, the formations of short chain fatty acids are 70 to 140 mmol L^−1^ and 20 to 70 mmol L^−1^, respectively [16]. Furthermore, smaller quantities of formate, caproate, valerate, 2-methyl-butyrate, and isovalerate are produced by microbial fermentation of prebiotics. However, in general, acetate is predominant, followed by proprionate and butyrate, formation of short-chain fatty acids depends on (a) molecular configuration of prebiotic (carbohydrate monomer, glycosidic linkage, and degree of polymerization); (b) interaction with gut bacteria; (c) saccharolytic capacities of probiotic synthesized enzymes; and (d) fermentation mechanism [17]. Imbalance of microbiota in the gut creates dysbiosis and is a risk factor for several health hazards [18,19].

Consumption of lactose-based prebiotics, together with probiotics, offers some advantages due to their symbiotic activity [6,20]. However, the proliferation of intestinal microflora is a gradual process with age, and lactose-based prebiotics support the growth of probiotics due to the presence of *bgal—LacS* operon, which encodes transporter protein, enzymes for lactose hydrolysis, and metabolism [17,21]. A wide range of symbiotic outcomes, such as the restoration of gut microbiota, the maintenance of an equilibrium of gut microbiota, prevention against risks of several health hazards, development of immunity against pathogens, neutralization of toxins, and synthesis of added-value metabolites are well documented [12]. Table 1 shows the biochemical mechanisms for the synthesis of lactose-based prebiotics and biological outcomes due to symbiosis of lactose-based prebiotics and probiotics.

Osteoporosis, hyperlipidemia, and hyperglycemia are common health hazards among all communities around the world. In adverse situations, they often cause death. Reduction of the risks of osteoporosis, hyperlipidemia, and hyperglycemia, by symbiosis of lactose-based prebiotics and probiotics, has been reported in several cases. In most cases, in vivo trials were conducted with animal and human models to verify the anti-osteoporosis, anti-hyperlipidemic, and anti-hyperglycemic effects of lactose-based prebiotics along with probiotics. These trials focused on outcomes, rather than understand the underlying biochemical mechanisms, which are therefore still unclear. Realizing the great potentialities of lactose-based prebiotics on human health, in this review article biochemical mechanisms and results of clinical investigations about controlling osteoporosis, blood- lipid, and glucose levels by lactose-based prebiotics and symbiosis with probiotics are reported in a comprehensive way. 

## 2. Osteoporosis

Osteoporosis is a common complication, characterized by porous bone, improper bone mass, and reduced bone- density and strength [22]. There is a profound relationship between gastrointestinal disease and osteoporosis. Osteoporosis is associated with (a) maldigestion and malabsorption of nutrients due to (a) celiac disease; (b) postgastrectomy; (c) short bowel syndrome; (d) inflammatory bowel diseases; (e) type 1 diabetes; (f) chronic liver disease; (g) gastroesophageal reflux disease; and (h) patients treated with total parenteral nutrition. Often patients suffer with osteoporosis after (a) liver and small bowel transplantation and (b) gastric bypass surgery [23,24]. Furthermore, overgrowth of gut microbiota, specifically Firmicutes, *Proteobacteria*, *Bacteroidetes* and *Actinobacteria* are also an important risk factor for osteoporosis [25,26].

### 2.1. Biochemical Mechanisms

**Figure 2:**
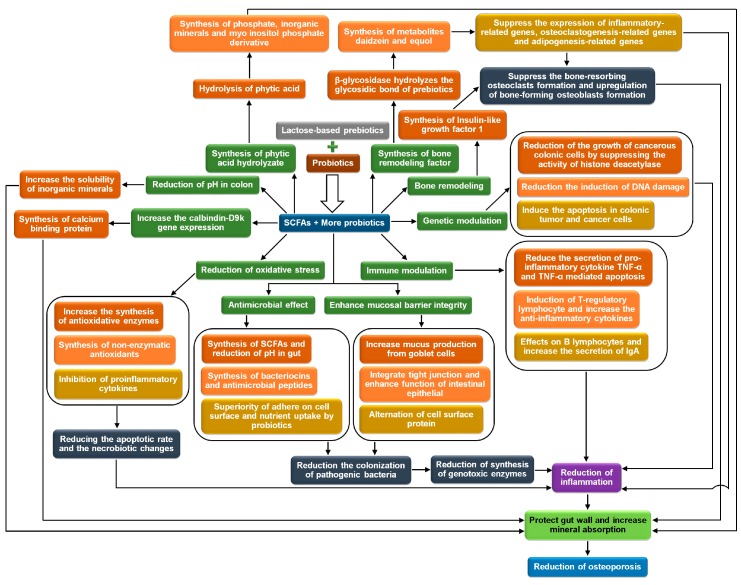
Osteoporosis reduction mechanisms, offered by lactose-based prebiotics and interaction with probiotics. SCFAs: Short-chain fatty acids; TNF-α: Tumor necrosis factor-α; IgA: Immunoglobulin A. (self-developed, figure compiled by authors based on Scholz-Ahrens et al., 2007 [31]; Whisner and Castillo, 2018 [32]; McCabe et al., 2015 [33]).

Lactose-based prebiotics or their interaction with probiotics reduce osteoporosis and improve bone health [27,28,29] via different mechanisms. The mechanisms are (a) transformation of insoluble inorganic salts to soluble by short-chain fatty acids and increase their absorption to the gut wall; (b) maintenance and protection of mineral absorption surface in the gut by promoting the proliferation of enterocytes and colonocytes, integrating gut epithelium cells and improving intestinal barrier defending activity, down-regulating the formation and activity of nuclear factor kappa-light-chain-enhancer of activated B cells, reducing oxidative stress, immunomodulation, genetic modulation, and increasing antimutagenic activity; (c) increase the expression of calcium-binding proteins in the gut wall by increasing calbindin-D9k gene expression; (d) remodel the formation of osteoclasts and osteoblasts by increasing calcium uptake by suppressing the activities of parathyroid hormone and synthesis of insulin-like growth factor 1; (e) release of bone modulating factors; and (f) degradation of mineral complexing phytic acid [30,31,32,33]. The involvement of several gut microbiota, including probiotics, such as *Butyricicoccus*, *Dialister*, *Oscillibacter*, *Lactobacillus*, *Lactococcus*, and Bifidobacteria reduce the risk of osteoporosis [34]. The detailed mechanisms of reduction of osteoporosis offered by lactose-based prebiotics and interaction with probiotics are presented in Figure 2 and subsequent sections.

#### 2.1.1. Conversion of Insoluble Inorganic Salts to Soluble and Their Absorption in the Gut

However, it has been reported that in the small intestine, dietary fibers may entrap or bind with inorganic minerals and inhibit their absorption. Fermented products of dietary fibers, i.e., short-chain fatty acids promote mineral absorption in the intestine [35]. Inorganic minerals are insoluble at a neutral pH [30]. Lactose-derivatives galacto-oligosaccharide [27,28] and lactulose [29] increase mineral absorption. In the intestine, lactose-based prebiotics are converted to short-chain fatty acids (butyric acid, acetic acid, and propionic acid) and lactic acid. As a result, the pH of the intestine reduces. In acidic pH, inorganic calcium [27,28,29], magnesium [29], iron [27], manganese [36], magnesium [37], boron [38], and copper and zinc [39] salts become soluble. This process promotes the colonic absorption of inorganic minerals to the gut. Inorganic minerals have play a significant role in the development of bone matrix constituents and they are essential cofactors for enzymes, involved in collagen synthesis [31,40].

#### 2.1.2. Maintenance and Protection of Mineral Absorption Surface Area in the Gut

Lactose-based prebiotics and probiotics work symbiotically to improve bone health (reducing bone loss) by protecting the mineral absorption surface in the gut [22,23,24]. In the intestine, prebiotic-derived undissociated- and dissociated- short-chain fatty acids are absorbed to microvilli by passive diffusion and active transport mechanisms, respectively. Lactate and butyrate are absorbed through G-protein coupled receptors GPR41 and GPR43, and act as a growth factor of enterocytes and colonocytes [41]. Moreover, probiotic synthesized polyamine acts as a luminal mucosal growth factor in the host [42,43]. Due to the proliferation and enlargement of absorption surface area in the gut, mineral absorptions are increased [30]. Furthermore, lactose-based prebiotics and probiotics protect and maintain gut wall surface (mineral absorption surface area) [44,45]. The mechanisms are (a) integration of gut epithelium cells through the synthesis of antimicrobial agents (short-chain fatty acids [46,47], bacteriocins [48,49], antimicrobial peptides [50,51], mucin [52,53], collagen, fibronectin or fibrinogen [54,55,56], bacterial s-layer protein [57,58,59], and lectin-like protein [60,61]), and improvement of intestinal mucosal barrier defending activity through the development of a mucus layer [53,62,63], integration of tight junction, and alternation of cell surface proteins [64,65,66]; (b) down-regulation of the formation and activity of nuclear factor kappa-light-chain-enhancer of activated B cells [67,68]; (c) reduction of oxidative stress by glutathione [69], superoxide dismutase [70,71], catalase [72], glutathione peroxidase type 2 [73], and peroxiredoxins [74]; (d) immunomodulation [75,76,77]; (e) genetic modulation [78,79,80]; and (f) increase of antimutagenic activity [81,82,83].

#### 2.1.3. Increase in the Activity of Calcium Binding Protein

Prebiotic-derived short-chain fatty acids, such as butyrate and propionate increase calbindin-D9k gene expression and are responsible for calcium binding protein synthesis via various mechanisms [84]. The probable mechanisms are (a) short-chain fatty acids directly enter cells and affect gene expression (inhibit the activity of histone deacetylase) [85,86] and (b) short-chain fatty acids bind with specific G-protein coupled receptors (GPR41 and GPR43), are present on cell membrane of the intestinal epithelial cells and affect calbindin-D9k transcription by intracellular signal transduction [87,88]. Upregulation of calcium binding protein increases bioavailability of key bone mineral, i.e., calcium within cells [89].

#### 2.1.4. Bone Remodeling

Short-chain fatty acids take part in bone remodeling (suppression of bone-resorbing osteoclasts formation and upregulation of bone-forming osteoblasts formation). An increase of calcium uptake is associated with a high level of bone accrual [90] and suppression of the activities of the parathyroid hormone as well as bone resorption [91,92]. Short-chain fatty acids influence the synthesis of insulin-like growth factor 1, which takes part in bone remodeling [93,94]. Insulin-like growth factor 1 can promote both bone resorption and formation via direct effects on osteoclasts [95] and osteoblasts [96,97], respectively. Moreover, local insulin-like growth factor 1 promotes bone growth and development in a significant way [98]. Lactose-based prebiotics support the growth of probiotics [99]. It has been reported that probiotic *Lactobacillus reuteri* ATCC PTA 6475 has the potential to suppress the activity of tumor necrosis factor α. It inhibits the Wnt10b RNA in osteoblasts when subjects have type 1 diabetes [100,101]. In another investigation, it has been suggested that *Lactobacillus reuteri* can suppress osteoclast activity in menopausal rodents [102]. Furthermore, *Lactobacillus reuteri* treatment has been shown to maintain bone health under low estrogen or estrogen-depleted conditions [102,103].

#### 2.1.5. Release of Bone Modulating Factors

Lactose-based prebiotics stimulate the growth of *Lactobacillus* and Bifidobacteria. They maintain the population of gut microflora [104], and their synthesized β-glycosidase hydrolyzes the glycosidic bond of prebiotics. Subsequently, metabolites daidzein and equol are produced in a consequent way [105]. Equol is a nonsteroidal estrogen and plays a significant role in bone maintenance. Specifically, equol suppresses the expression of inflammatory-, osteoclastogenesis- and adipogenesis-related genes [106]. Under estrogen deficient conditions, galacto-oligosaccharide prevents bone loss in ovariectomized rats and mice [107]. Furthermore, the formation of equol from lactulose has been reported by several research groups [108,109].

#### 2.1.6. Degradation of Mineral Complexing Phytic Acid

Humans and monogastric animals cannot produce endogenous phytase, which is responsible for the degradation of phytic acid, and is present as phytate form in cereal foods [110]. In enzymatic conversion, phytase hydrolyzes phosphomonoester bonds in phytate and transforms into an inorganic phosphate and a myo inositol phosphate derivative [111]. In the intestine, lactose-based prebiotics enhance the survival of probiotics and their activities [99]. They have the potential to synthesize phytase due to the presence of *appCBA* operon [112]. Consumption of prebiotics, along with probiotics, increases the bioavailability of trace elements, such as phosphate, copper, zinc, and iron. They play a significant role in bone development and the suppression of osteoporosis [113,114].

### 2.2. Clinical Investigations

Some clinical investigations have been performed with different types of lactose-based prebiotics, such as galacto-oligosaccharide and lactulose, to understand their effectiveness on mineral absorption. A randomized crossover study was performed with 12 healthy non-anemic males, aged 20 to 30 years, to investigate the effect of galacto-oligosaccharide on true intestinal absorption of iron and calcium. A double stable-isotope technique was adopted for experimental purposes. The subjects consumed a controlled basal diet supplemented with 15 g day^−1^ inulin or fructo-oligosaccharide or galacto-oligosaccharide or without non-digestible oligo-saccharide (control diet). During the first 2 weeks of each type of diet, subjects were in a normal environment. On days 15 to 21 (last week) of each treatment, members consumed 0.05 g of non-digestible oligo-saccharide with orange juice at the start of breakfast, lunch, and dinner. Oral administration of ^57^Fe and ^44^Ca and intravenous ^58^Fe and ^48^Ca were used in the investigation. Iron absorption was measured on days 15 to 21 (the last 7 days of treatment) and calcium absorption was measured on day 21. It was found that there were no significant differences in calcium and iron absorptions. The authors concluded that 15 g day^−1^ prebiotic treatment had no negative effect on iron and calcium absorptions in subjects [27]. van den Heuvel et al., performed a double-blind randomized crossover investigation with 12 post-menopausal women (mean age 62 years) to understand the function of galacto-oligosaccharide on true calcium absorption in mucosa. The experimental schedule consisted of two 9-days treatment periods, separated by a 19-day washout period. During first period, seven subjects received reference treatment (sucrose supplemented yogurt) and six subjects received 200 mL of yogurt twice a day (at breakfast and lunch) containing galacto-oligosaccharide 100 g L^−1^. In the second period, the study protocol was reversed. During treatment periods, subjects maintained their habitual food consumption and excluded the consumption of prebiotic- or probiotic- containing products. On the 8th day of each treatment period, ^48^Ca and ^44^Ca were administered intravenously and orally, respectively. It was found that the mean calcium absorption level was high in galacto-oligosaccharide yogurt-treated group compared to the control group. Moreover, total calcium excretion in urine after 36 h was low in galacto-oligosaccharide yogurt-treated group compared to the control group [28]. Another double-blind randomized, crossover investigation was performed by Seki et al., with 24 healthy adult male volunteers (mean age 33.5 ± 5.5 years). Test foods, containing 4 g (high-dose) or 2 g (low-dose) of lactulose together with 150 mg of magnesium and 300 mg of calcium were administered orally. In test food, 28 mg of ^25^Mg and 20 mg of ^44^Ca were present in 150 mg of magnesium and 300 mg of calcium, respectively. The subjects were randomly divided into three groups (*n* = 8 in each group), designated as group A, group B, and group C. At first ingestion period, members of group A, group B, and group C received a placebo formula, low-dose lactulose formula, and high-dose lactulose formula, respectively. After a wash out period of 2 weeks, in the second ingestion period, members of group A, group B, and group C received low-dose lactulose formula, high-dose lactulose formula, and a placebo formula, respectively. Subsequently, there was a 2-week washout period and in the third ingestion period, members of group A, group B, and group C received high-dose lactulose formula, a placebo formula, and low-dose lactulose formula, respectively. Concentrations of isotope ions were measured in urine samples. It was found that the least-square mean of urinary stable-isotopes ratios (^44^Ca/^40^Ca and ^25^Mg/^24^Mg) were increased in a dose-dependent manner. Significant differences in calcium and magnesium ratios between placebo-, low dose-, and high dose-lactulose-treated subjects were observed, and changes in Ca/creatinine and Mg/creatinine had similar trends [29].

## 3. Controlling Blood Lipids

Cardiovascular disease is the result of hyperlipidemia or dyslipidemia in elderly individuals. High levels of low-density lipoprotein cholesterol, triglyceride-rich lipoproteins, and low levels of high-density lipoprotein cholesterol in circulatory system are widely recognized risk factors for cardiovascular diseases (atherosclerosis, coronary heart diseases), which may be the result of consumption of an unhealthy diet containing high amounts of fat, salts, and simple carbohydrates [115]. It has been reported that the risk of a heart attack is three times higher in hypercholesterolemic patients, compared to normal individuals [116]. Different types of hyperlipidemia are primary hyperlipoproteinemia, polygenic hypercholesterolemia, familial combined hyperlipidemia, familial dysbetalipoprotenemia, familial hypertriglyceridemia, and endogenous hypertriglyceridemia [117]. Risk factors for hyperlipidemia in individuals includes (a) high age; (b) sex (generally men suffer with coronary heart disease and women may suffer after menopause); (c) family history; (d) type 2 diabetes or insulin resistance; (e) above average weight or obesity; (f) high cholesterol and triglycerides accumulation in blood transportation system, and consequently high blood pressure; (g) sleep apnea; (h) presence of high sensitivity C-reactive protein; (i) high level of homocysteine; (j) preeclampsia during pregnancy; (k) autoimmune diseases (rheumatoid arthritis and lupus); (l) high stress; (m) smoking; (n) consumption of alcohol and unhealthy diet; and (o) low physical activity and sedentary lifestyle [118,119]. Overgrowth of gut microbiota, such as *Eggerthella*, *Akkermansia*, *Christensenella*, *Tenericutes*, *Pasteurellaceae*, and *Butyricimonas* are inversely correlated with serum triglyceride and positively associated with serum high-density lipoprotein cholesterol, risk factors of hyperlipidemia or dyslipidemia in individuals [120,121,122].

### 3.1. Biochemical Mechanisms

**Figure 3:**
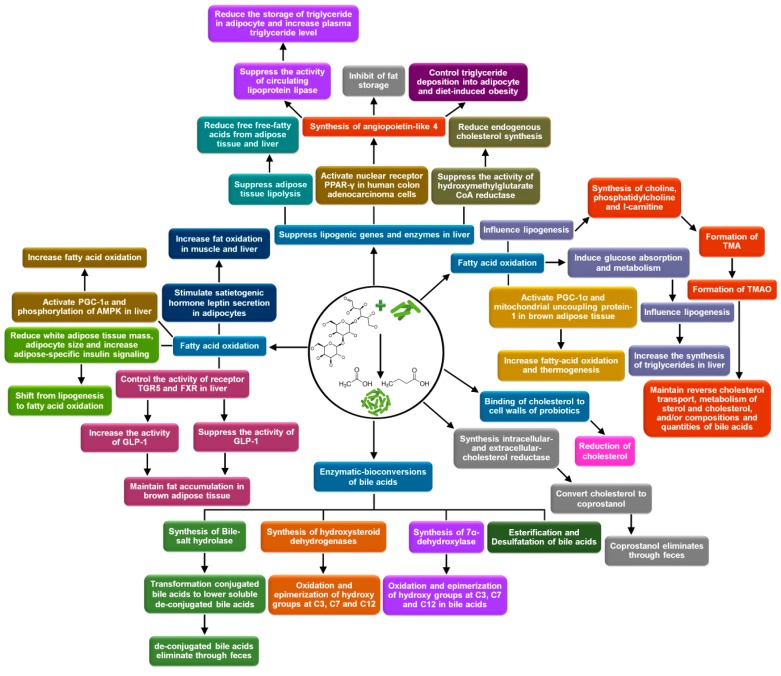
Blood lipid controlling mechanisms, offered by lactose-based prebiotics and interaction with probiotics. SCFAs: Short-chain fatty acids; TMA: Trimethylamine; TMAO: Trimethylamine N-Oxide; PGC-1α: Peroxisome proliferator-activated receptor gamma coactivator 1α; PPAR-γ: Peroxisome proliferator-activated receptor-γ; AMPK: Adenosine monophosphate-activated protein kinase; FXR: Nuclear farnesoid X receptor; GLP-1: Glucagon-like peptide-1. (self-developed, figure compiled by authors based on Kumar et al., 2012 [129]; Anandharaj et al., 2014 [115]; Kasubuchi et al., 2015 [130]).

Lactose-based prebiotics or their interaction with gut microbiota and probiotics control lipid level in bloodstream and tissue [123,124]. Different biochemical mechanisms have been reported in this context. The mechanisms are (a) suppression of lipogenic genes expression as well as the activities of lipogenic enzymes; (b) oxidation of fatty acids; (c) the binding of cholesterol to the cell walls of probiotics and their assimilation; (d) enzymatic-conversions (de-conjugation, oxidation, and epimerization of hydroxyl groups at C3, C7, and C12, 7-dehydroxylation, esterification, and desulfatation) of bile acids; and (e) conversion of cholesterol to coprostanol and its defecation [125,126]. In the intestine, several consortia, such as *Bacteroides*, Bifidobacteria, *Clostridia*, *Lactobacillus*, *Listeria*, *Egghertella*, *Eubacteria*, *Peptostreptococcus*, *Ruminococcus*, *Fusobacteria*, *Peptococcus*, and *Pseudomonas* play a role in the above mentioned biochemical reactions [122,127,128]. The detailed mechanisms of controlling blood lipid level offered by lactose-based prebiotics and interaction with probiotics are presented in Figure 3 and subsequent sections.

#### 3.1.1. Suppression of Lipogenic Gene Expression and Activities of Lipogenic Enzymes

Short-chain fatty acids reduce the synthesis of cholesterol, fatty acid, triacylglycerol, and very-low-density lipoprotein via suppression of lipogenic gene expression. They also reduce the activities of lipogenic enzymes (acetyl-CoAcarboxylase, malic enzyme, fatty acid synthase, ATP citrate lyase, and glucose-6-phosphate dehydrogenase) in the liver [131]. Also in the liver, acetate is converted to acetyl CoA and acts as a lipogenic substrate for *de novo* lipogenesis, whereas propionate inhibits lipid synthesis [132,133]. A high level of circulating short-chain fatty acid is linked with reduced adipocyte lipolysis and adipogenesis [134]. Suppression of adipose tissue lipolysis supports the reduction of free-fatty acids in adipose tissue and the liver [135]. Furthermore, involvement of lactose-based prebiotics and probiotics suppress the activity of hydroxymethylglutarate CoA reductase as well as endogenous cholesterol synthesis [136]. Short-chain fatty acids, mainly acetic acid, propionic acid, and butyric acid stimulate the synthesis of intestinal fasting-induced adipocyte factor, such as angiopoietin-like 4, by activating the peroxisome proliferator activated receptor γ in human colon adenocarcinoma cells [137,138] and subsequently inhibit fat storage. Probiotics increase the synthesis of angiopoietin-like 4, which leads to suppression of the activity of circulating lipoprotein lipase [139] and consequently reduces the storage of triglyceride in adipocyte and increases plasma triglyceride level [140,141]. Furthermore, angiopoietin-like 4 controls triglyceride deposition to adipocyte and diet-induced obesity [140,141,142].

#### 3.1.2. Fatty Acid Oxidation

In muscle and liver tissue, butyrate enhances fatty acid oxidation by increasing the expression of peroxisome proliferator-activated receptor-gamma coactivator-1α and phosphorylation of adenosine-monophosphate-activated kinase [143,144]. In brown adipose tissue, butyrate enhances thermogenesis and fatty acid oxidation by increasing the expression of peroxisome proliferator-activated receptor-gamma coactivator-1α and mitochondrial uncoupling protein-1 [145,146]. Short-chain fatty acids influence bile acid receptors, such as membrane-bound G-protein coupled receptor TGR5 and nuclear farnesoid X receptor, and suppress fat accumulation in brown adipose tissue. Receptor TGR5 induces glucagon-like peptide-1 synthesis [147], whereas activation of receptor nuclear farnesoid X receptor reduces its activity [148]. Short-chain fatty acids reduce white adipose tissue mass and adipocyte size, and increase adipose-specific insulin signaling [149,150]. These promote a shift from lipogenesis to fatty acid oxidation [134,150]. Furthermore, short-chain fatty acids stimulate satietogenic hormone leptin secretion in adipocytes. Leptin increases fat oxidation in both muscle and liver tissue [151,152]. Gut microbiota increase the synthesis of triglycerides in the liver. Sterol response element binding protein 1c, carbohydrate response element binding protein, acetyl-CoA carboxylase, fatty acid synthase, and adenosine 5′-monophosphate–activated protein kinase influence lipogenesis through inducing glucose absorption and metabolism as well as insulin level [153]. Furthermore, intestinal microbiota produce trimethylamine N-oxide through the oxidation of trimethylamine by flavin monooxygenase in liver. Trimethylamine is a microbial product, derived from choline, phosphatidylcholine, and l-carnitine. Trimethylamine N-oxide reduces the risks of atherosclerosis and cardiometabolic through the perturbations of reverse cholesterol transport, metabolism of sterol and cholesterol, and/or compositions and quantities of bile acids [122].

#### 3.1.3. Binding of Cholesterol to the Cell Walls of Probiotics and Their Assimilation

Lactose-based prebiotics endorse the growth of probiotics, and these offer an anti-hyperlipidemic effect to the host [115,126]. Cholesterol can bind with the cell membranes of probiotics [154,155]. Bile-salt hydrolase supports the incorporation of cholesterol into the cell membranes of probiotics [156,157]. In probiotic cell membranes, cholesterol accumulates in the regions of phospholipid tails, upper phospholipids, and polar heads of the membrane phospholipid bilayer [158,159]. It has been proven that growing cells, dead cells, and heat-killed probiotic cells are able to reduce cholesterol levels [158,159,160], and cholesterol removal is higher with growing cells than dead cells and heat-killed cells [161,162]. Assimilation of cholesterol into cellular membrane alters the fatty acid composition in cells. High-level accumulation of fatty acids (both unsaturated- and saturated-fatty acids) in cells leads to stronger membrane and cellular resistance, and subsequently increases the possibility of cell lysis [158,159].

#### 3.1.4. Enzymatic-Conversions of Bile Acids

Lactose-based prebiotics promote the growth and activities of gut microbiota as well as probiotics [99]. Biotransformations of bile acids are involved with intestinal microorganisms, including probiotics during their enterohepatic circulation. Microbial bioconversions of bile acids include (a) de-conjugation, (b) oxidation and epimerization of hydroxy groups at C3, C7, and C12, (c) 7α/7β-dehydroxylation; and (d) esterification and desulfatation [127,128].

##### De-Conjugation

In the large intestine, certain facultative and anaerobic consortia, including probiotics (*Bacteroides*, *Lactobacillus*, Bifidobacteria, *Clostridia*, and *Listeria*) produce secondary bile acids (taurocholic acid, glycocholic acid, taurochenodeoxycholic acid, and glycochenodeoxycholic acid) from the pool of bile acids, such as cholic acid and chenodeoxycholic acid [163]. Intestinal microbes can alter the amount and type of secondary bile acids via nuclear farnesoid X receptor and TGR5 signaling. Bile-salt hydrolase from intestinal microbiota and probiotics hydrolyzes conjugated glycodeoxycholic acid and taurodeoxycholic acid in enterohepatic circulation and produces lower soluble de-conjugated bile acids (de-conjugation of glycol- and tauro-bile acids). A small fraction of these bile acids are absorbed by passive diffusion to the small intestine, and provide active transportation to the ileum and passive absorption to the colon [164]. As a consequence, they are eliminated via feces. Cholesterol is used to produce new bile acids to maintain homeostasis, which reduces the physiological cholesterol pool in the bloodstream [165]. In the intestine, some bacterial species use residual end products (carbon, nitrogen, and sulfur) of bile acids de-conjugation [128,166].

##### Oxidation and Epimerization of Hydroxy Groups at C3, C7, and C12

Gut microbiota catalyze oxidation/reduction of hydroxy groups at the 3-, 7-, and 12-carbons of bile acids with hydroxysteroid dehydrogenases. Epimerization of hydroxy groups occurs via stereospecific oxidation and, subsequently, stereospecific reduction with α-hydroxysteroid dehydrogenases and β-hydroxysteroid dehydrogenases, respectively [128,166]. The formation of stable oxo-bile acid intermediate is influenced by the catalytic activity of bacterial hydroxysteroid dehydrogenases, pH of environment and presence of pyridine nucleotides. Generally, both 3α-hydroxysteroid dehydrogenase and 3β-hydroxysteroid dehydrogenase are present in Firmicutes. However, 7α-hydroxysteroid dehydrogenases are present in *Eubacterium*, *Clostridia*, and *Bacteroides*, 7β-hydroxysteroid dehydrogenase is only present in Firmicutes. Furthermore, 12α-hydroxysteroid dehydrogenase and 12β-hydroxysteroid dehydrogenase have been discovered in different Firmicutes [128,164]. The epimerization of bile acids reduces the toxicity of hydrophobic chenodeoxycholic acid to intestinal microorganisms [164,167].

##### 7α/7β-Dehydroxylation

Due to the unavailability of hydroxyl group after deconjugation of conjugated bile acids with bile-salt hydrolase, dehydroxylation of primary bile acids (cholic acid and chenodeoxycholic acids) takes place. Involvement of multiple genes in *bai* operon, removal of 7α-hydroxy or 7β-hydroxy group from primary bile acids, produces deoxycholic and lithocholic acids [166]. Activity of 7α-dehydroxylase has been identified in *Eubacterium* and *Clostridium* [128].

##### Esterification and Desulfatation

Intestinal microbiota produce esters of bile acids by esterification of C-24 carboxyl group of molecule associate with 3α-hydroxy group of the neighbor one in bile acids. Mixed fecal consortia are responsible for these bioconversions. Generally, *Bacteroides*, *Lactobacillus*, and *Eubacteria* participate in esterification of bile acids and *Clostridia*, *Fusobacteria*, *Peptococcus*, and *Pseudomonas* participate in desulfatation of bile acids [128,164].

#### 3.1.5. Conversion of Cholesterol to Coprostanol and Its Defecation

Cholesterol levels in human individuals are balanced by lipid absorption, metabolic, and excretion processes. They are influenced by gut microflora, including probiotics [128,168]. In the intestine, lactose-based prebiotics stimulate the growth of probiotics [99] that convert cholesterol to coprostanol. Coprostanol is not water-soluble and is poorly absorbed in the gut [168], which promotes its direct defecation. Cholesterol dehydrogenase/isomerase produced by cholesterol oxidizing bacteria catalyzes the transformation of cholesterol to coprostanol [169]. Probiotics, synthesized by intracellular- and extracellular-cholesterol reductase, convert cholesterol to coprostanol [159]. There are two major pathways for the conversion of cholesterol to coprostanol that have been reported. In the first pathway, direct reduction of the 5 to 6 double bond in cholesterol takes place. In the second pathway, primarily oxidation of the 3β-hydroxy group and isomerization of double bond to form of 4-cholesten-3-one take place. Subsequently, coprostanone and then coprostanol are formed through the reduction pathway [128,170]. A decrease in the amount of cholesterol via enzymatic conversion and subsequently defecation leads to reduction of physiological cholesterol pool [127,128].

### 3.2. Clinical Investigations

Some clinical investigations have been performed with different types of lactose-based prebiotics to understand their anti-hyperlipidemic activities. Vogt et al., performed a semi-randomized crossover study with 18 healthy men (age 18 to 60 years) to understand the effects of lactulose on blood lipid profile and colonic short-chain fatty acids on hepatic lipid metabolism. The subjects were divided into two groups. The first group (*n* = 9) was randomly assigned to consume either L-rhamnose or lactulose 25 g day^−1^. After a 3-month washout period, they enrolled in a D-glucose study period and among them seven subjects participated in a third study period. During study period three, they consumed the remaining lactulose or L-rhamnose. Subjects in the second group (*n* = 9) consumed D-glucose in their first study period and the day after the first study period, they were randomly assigned to consume either lactulose or L-rhamnose for a second study period. After a 6-month washout period, all subjects from second group and two from first group completed their final study period. It was reported that the sugar type did not affect fasting total cholesterol and triglyceride levels on either initial day or after 4 weeks, log-transformed values of fractional synthetic rates for triacylglycerol-fatty acids were significantly lower for the L-rhamnose-treated group and the lactulose-treated group than the D-glucose-treated group. In a similar way, absolute synthetic rates for triacylglycerol-fatty acids were lower for the L-rhamnose-treated group and the lactulose-treated group than the D-glucose-treated group [123]. Another double-blind, placebo controlled trial experiment was performed with 45 human subjects (male, *n =* 16, age 42.8 ± 12.1 years; female, *n =* 29, age 46.4 ± 11.8 years), who presented some risk factors of metabolic activities (fasting glucose 5.5 ± 0.8 mmol L^−1^, total cholesterol 6.6 ± 1.2 mmol L^−1^, high density lipid cholesterol 1.2 ± 0.2 mmol L^−1^ and triglyceride 2.1 ± 0.9 mmol L^−1^ in men; fasting glucose 5.2 ± 0.6 mmol L^−1^, total cholesterol 6.1 ± 1.3 mmol L^−1^, high density lipid cholesterol 1.5 ± 0.3 mmol L^−1^ and triglyceride 1.3 ± 0.4 mmol L^−1^ in women). Subjects were randomly assigned to a placebo group, a group treated with maltodextrin, and experimental, a group treated with galacto-oligosaccharide. Members of individual groups consumed respective products at 5.5 g day^−1^ for 12 weeks, followed by a washout period of 4 weeks, before switching to another intervention for a final 12 weeks. It was reported that the concentrations of high density lipid cholesterol and low density lipid cholesterol in plasma were unchanged in members of both the placebo group and the experimental group, and the total concentration of cholesterol in plasma after the 12-week experiment was significantly lower in the galacto-oligosaccharide-treated group than the placebo group [124].

## 4. Controlling Blood Glucose Level

Of all different kinds of diabetes (type 1 diabetes, type 2 diabetes, gestational diabetes, latent autoimmune diabetes of adults, maturity onset diabetes of the young, and neonatal diabetes), type 2 diabetes is the most common, resulting in cardiovascular disease, retinopathy, nephropathy, neuropathy, hearing damage, skin damage, leg ulcers, and gangrene [171,172]. Insulin from islets of Langerhans in the pancreas plays a crucial role in the regulation of glucose homoeostasis as well as blood glucose level [173,174]. Failure of response to insulin is the primary cause of type 2 diabetes. Excess accumulation of visceral fat causes chronic low-grade inflammation, regarded as a high level of macrophage infiltration. Furthermore, accumulation of visceral fat promotes insulin resistance and compensatory hyperinsulinemia [175,176]. In peripheral tissues, pro-inflammatory adipokines hinder insulin signaling and may cause insulin resistance [177,178,179]. The risk factors for type 2 diabetes include (a) obesity; (b) age; (c) sex; (d) heredity; (e) hypertension; (f) Alzheimer’s disease; (g) smoking; and (h) sedentary lifestyle [41,130,177,180,181]. Gut microbiota have direct correlation with both type 1 diabetes and type 2 diabetes. Type 2 diabetes is associated with the declination of *Roseburia*, *Faecalibacteria*, *Clostridia*, *Betaproteobacteria*, and *Eubacteria*, and an increase of several pathogens, such as *Bacteroides*, *Akkermansia*, *Desulfovibrio*, and some pathogenic *Clostridia* [120,182]. Type 1 diabetes is associated with the reduction of Firmicutes, *Lactobacillus*, Bifidobacteria, *Blautia*, *Eubacteria*, and *Prevotella*, and an increase of *Bacteroidetes*, pathogenic *Clostridia*, and *Veillonella* [182].

### 4.1. Biochemical Mechanisms

**Figure 4:**
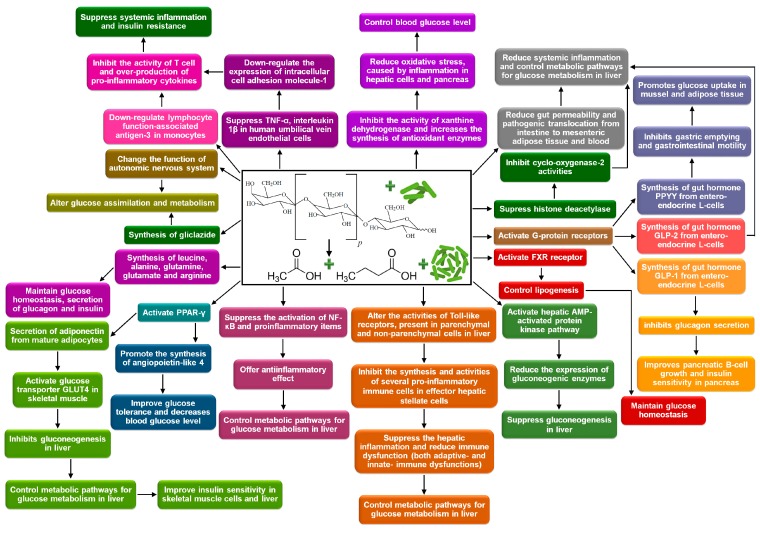
Hyperglycemia controlling mechanisms, offered by lactose-based prebiotics and interaction with probiotics. TNF-α: Tumor necrosis factor-α; PPAR-γ: Peroxisome proliferator-activated receptor-γ; AMPK: Adenosine monophosphate-activated protein kinase; GLP-1: Glucagon-like peptide-1; GLP-2: Glucagon-like peptide-2; NF-κB: Nuclear factor- κB. (self-developed, figure compiled by authors based on Kasubuchi et al., 2015 [130]; Janssen and Kersten, 2015 [181]; Sáez-Lara et al., 2016 [188]).

Research has proven that lactose-based prebiotics reduce long-standing high levels of blood glucose (hyperglycemia) in hosts [183,184]. The mechanisms include: (a) Controlling the synthesis and activities of gut hormones by synthesis of gut hormones plasma peptide-YY, glucagon-like peptide-1 and glucagon-like peptide-2; (b) altering glucose assimilation and metabolism through protecting the liver from inflammation, control of gluconeogenesis, synthesis of angiopoietin-like 4 and activation of peroxisome proliferator-activated receptor γ; (c) controlling the synthesis and activities of pro-inflammatory and anti-inflammatory cytokines through immunomodulation; (d) reducing oxidative stress through suppressing inflammation and enhancing intestinal barrier, different antioxidative mechanisms, such as reactive oxygen species scavenging, metal ion chelation, down-regulated ascorbate autoxidation, and synthesis of antioxidant enzymes and molecules; and (e) producing amino acids [41,177,180,185]. Involvement of several beneficial intestinal microbes, such as *Lactobacillus*, Bifidobacteria, *Propionibacteria*, *Clostridia*, *Akkermansia*, and *Faecalibacteria* provide protection from diabetes [186,187]. The detailed mechanisms regarding reduction of hyperglycemia, offered by lactose-based prebiotics and interaction with probiotics are presented in Figure 4 and subsequent sections.

#### 4.1.1. Control the Synthesis and Activities of Gut Hormones

In the intestine, lactose-based prebiotics are converted to short-chain fatty acids (butyric acid, acetic acid, and propionic acid) and lactic acid by probiotics [99]. They increase the synthesis of gut hormones plasma peptide-YY, glucagon-like peptide-1 and glucagon-like peptide-2 from entero-endocrine L cells through the activation of G-protein coupled receptors [130,189]. Glucagon-like peptide-1 inhibits glucagon secretion and impedes gluconeogenesis in the liver. Furthermore, it improves B-cell growth and insulin sensitivity in the pancreas [177]. Plasma peptide-YY inhibits gastric emptying and gastrointestinal motility, and promotes glucose uptake in muscle and adipose tissue [190,191]. It has been proven that propionate increases postprandial plasma peptide-YY and glucagon-like peptide-1 concentrations [153]. Butyrate improves insulin sensitivity [146] and acetate improves glucose tolerance [192]. Furthermore, it has been reported that probiotics increase the activities of gut hormone glucagon-like peptide-2, and decrease intestinal permeability, hepatic inflammation, and oxidative stress associated with the risk of diabetes [193].

#### 4.1.2. Alternation of Glucose Assimilation and Metabolism

Lactose-based prebiotics associate with probiotics maintain glucose homeostasis. In the body, glucose homeostasis is maintained by glucose assimilation and metabolism. Symbiosis of lactose-based prebiotics and probiotics reduces immune dysfunction (both adaptive- and innate-immune dysfunctions) by suppressing hepatic inflammation and controlling several metabolic pathways of glucose metabolism in the liver. Suppression of immune dysfunction is associated with alternations of the activities of Toll-like receptors, present in parenchymal and non-parenchymal cells in the liver. The alternation of the activities of Toll-like receptors controls the synthesis and activities of several pro-inflammatory immune cells (interleukin 6, interleukin 18, and other pro-inflammatory mediators) in effector hepatic stellate cells [194,195]. Butyrate and propionate reduce gluconeogenesis in the liver through the activation of hepatic adenosine monophosphate-activated protein kinase pathway, which decreases the gene expressions of gluconeogenic enzymes, such as glucose 6-phosphatase and phosphoenolpyruvate carboxykinase [144,150], associated with affecting gut–brain neural circuitry [196,197]. Short-chain fatty acids (butyric acid, acetic acid, and propionic acid) promote the synthesis of angiopoietin-like 4, which improves glucose tolerance and decreases blood glucose [198]. Activity of angiopoietin-like 4 is induced by activation of peroxisome proliferator-activated receptor γ. Transcription factor peroxisome proliferator-activated receptor γ improves adiponectin secretion from mature adipocytes, which activates glucose transporter GLUT4 in skeletal muscle [199,200] and subsequently inhibits gluconeogenesis in the liver [201]. This process leads to improved insulin sensitivity in skeletal muscle cells and the liver. Furthermore, probiotics may alter glucose assimilation and metabolism by delaying or inhibiting glucose absorption in the intestine, increasing bioavailability of gliclazide [202] and changing the function of autonomic nervous system [203]. Gut microbiota control glucose absorption and metabolism, and subsequently lipogenesis in the liver through regulating sterol response element binding protein 1c, carbohydrate response element binding protein, acetyl-CoA carboxylase, fatty acid synthase, and adenosine 5′-monophosphate–activated protein kinase [204]. Intestinal microbiota influence the synthesis and concentrations of bile acids, which control glucose homeostasis through the activation of the nuclear farnesoid X receptor and membrane-bound G-protein coupled receptors [205]. In the ileum, activation of nuclear farnesoid X receptor controls the synthesis of fibroblast growth factor-19, which affects glucose tolerance [206]. In the pancreas, activation of nuclear farnesoid X receptor controls insulin transport and secretion [207]. In the liver, activation of nuclear farnesoid X receptor improves insulin sensitivity [208]. In the ileum, activation of G-protein coupled receptors control the production of glucagon-like peptide-1, which plays a role in the maintenance of glucose homeostasis [177]. 

#### 4.1.3. Immunomodulation

Alterations of hepatic natural killer T-cell activity might lead to relative over-production of pro-inflammatory cytokines, which is the cause of systemic inflammation and insulin resistance [177,209]. The pro-inflammatory tumor necrosis factor-α/inhibitor of nuclear factor kappa-B kinase subunit beta signaling pathway mediates insulin resistance [210,211]. Activation of inhibitor of nuclear factor kappa-B kinase subunit beta causes both hepatic and systematic insulin resistance [212]. Lactose-based prebiotics are converted to short-chain fatty acids and promote the growth of probiotics, and these offer an anti-diabetic effect via prevention of inflammation and immunomodulation. Symbiosis of lactose-based prebiotics and probiotics reduces gut permeability as well as pathogenic translocation from the intestine to mesenteric adipose tissue and blood. Consequently, these reduce systemic inflammation [213,214]. Short-chain fatty acids offer anti-inflammatory effects by inhibiting the activation of nuclear factor kappa-light-chain-enhancer of activated B cells (a transcriptional factor) [215,216] as well as suppressing proinflammatory items, such as high-sensitivity C-reactive protein, tumor necrosis factor α, interferon γ, interleukin 1β, and interleukin 6 [217,218,219]. Short-chain fatty acids suppress the activities of tumor necrosis factor α and nuclear factor kappa-light-chain-enhancer of activated B cells by facilitating PGE2 levels and cyclo-oxygenase-2 activities by inhibiting histone deacetylase [216,220]. Furthermore, butyrate decreases the expression of chemokine MCP-1 [217]. Butyrate inhibits the activity of T cells by down-regulating the expression of intracellular cell adhesion molecule-1 through the suppression of tumor necrosis factor α and interleukin 1β in human umbilical vein endothelial cells [221,222], and lymphocyte function-associated antigen-3 in monocytes [223]. Several intestinal bacteria induce the formation of inflammatory T cells (T helper 1 cell and T helper 17 cell) as well as synthesis of interleukin 1, interleukin 6, and interleukin 12. Furthermore, commensal microbiota stimulate the expression of FOXP3 (scurfin) in CD4 + T cells and differentiation of T_reg_ cells, those lead to the production of secretory immunoglobulin A [224].

#### 4.1.4. Reduction of Oxidative Stress

Lactose-based prebiotics are converted to lactic acid, short-chain fatty acids and promote the growth of probiotics. Short-chain fatty acids associated with probiotics may reduce oxidative stress (over production of reactive oxygen species and reactive nitrogen species), caused by inflammation in hepatic cells and the pancreas, and may control glucose level in the blood [189,225,226]. Short-chain fatty acids promote antioxidation and decrease oxidative stress in diabetic patients [227]. Butyrate inhibits the activity of xanthine dehydrogenase and increases the synthesis of glutathione, an antioxidant. Furthermore, they suppress purine catabolism as the formation of uric acid and reactive oxygen species [69]. Prebiotic-derived short-chain fatty acids suppress the synthesis and activities of pro-inflammatory cytokines, chemokines, and pro-inflammatory mediators, generated during oxidative stress [218]. Probiotics involved in several biochemical mechanisms, such as protection from inflammation in the gut and destruction of tight junctions, reactive oxygen species scavenging, metal ion chelation, and down-regulated ascorbate autoxidation [225,226]. Probiotics suppress the inflammation by reducing translocation of pathogens through enhancing intestinal barrier by (a) integration of gut epithelium cells through the synthesis of short-chain fatty acids [46,47], bacteriocins [48,49], antimicrobial peptides [50,51], mucin [52,53], collagen, fibronectin or fibrinogen [54,55,56], bacterial s-layer protein [57,58,59], and lectin-like protein [60,61]; (b) superiority of probiotics to adhere to mucosal surface; and (c) improvement of intestinal mucosal barrier defending activity through the development of a mucus layer [53,62,63] and integration of tight junction and alternation of cell surface proteins [64,65,66]. The pattern recognition toll-like receptors in gut epithelial layer recognize probiotic signals and bind them with lectin-like proteins. Subsequently, nuclear factor kappa B deactivates the expression of pro-inflammatory cytokine, such as tumor necrosis factor α and immune regulatory cytokine interferon γ [228]. Antioxidative enzymes, such as superoxide dismutase, catalase, glutathione peroxidase type 2, and peroxiredoxins from probiotics play role in reducing oxidative stress. Furthermore, probiotics inhibit oxidative stress through the synthesis of non-enzymatic antioxidants, such as glutathione, folate, and exopolysaccharide [225,226].

#### 4.1.5. Bioavailability of Amino Acids

In the gastrointestinal tract, lactose-based prebiotics endorse the growth of probiotics and maintain equilibrium of intestinal microbiota. In the intestine, proteins are hydrolyzed to peptides and amino acids by host- and bacterial-peptidases and proteases, and both gut bacteria and the host further utilize synthesized peptides and amino acids. Synthesized amino acids are incorporated to host- and bacterial-cells as building block of protein. The preferential amino acids for intestinal microbiota are lysine, glycine, arginine, valine, isoleucine, and leucine [229] and generate a complex mixture of metabolic end products, i.e., lactic acid, short-chain fatty acids (butyric acid, acetic acid, and propionic acid), branched-chain fatty acids (isobutyric acid, valeric acid, and isovaleric acid), and ammonia. It has been reported that undigested proteins and amino acids in the colon may serve as an additional substrate for production of short-chain fatty acids [230,231]. Furthermore, several amino acids produced by protein fermentation can serve as precursors for the synthesis of short-chain fatty acids [232]. It has been reported that amino acids play a role in the maintenance of glucose homeostasis as well as secretion of glucagon and insulin [233]. However, higher concentrations of brunch-chain amino acids in blood are associated with risks of developing type 2 diabetes [234], leucine, alanine, glutamine, glutamate, and arginine stimulate β-cell activity and insulin secretion [235].

### 4.2. Clinical Investigations

In this context, some clinical investigations have been performed with different lactose-based prebiotics. Mooradian et al., performed an experiment with 48 male subjects, (mean age 52.4 ± 1.8 years), who suffered with diabetes mellitus and, among them, 14 subjects were treated with insulin therapy. Mean fasting plasma glucose was 200 ± 14.4 mg dL^−1^ and mean glycosylated haemoglobin was 10.3 ± 0.33%. Thirteen diabetic patients had clinically significant renal disease (proteinuria greater than a trace or creatinine greater than 1.3). For all patients, serum creatinine level was not greater than 2.0. Results were compared with 13 males, aged between 27 to 62 years, had normal fasting plasma glucose, and glycosylated haemoglobin levels (considered as the control). Subjects consumed an oral sugar solution (20 g of sucrose, 1 g of L-rhamnose, 20 g of lactose and 5 g of lactulose were added in 7.5 cc Cephulac in a volume of 110 cc) within a period of 3 min followed by the consumption of an equal volume of water after an overnight fast. Patients maintained fasting for additional 2 h and subsequently consumed water ad libidum to produce a sufficient amount of urine. It was reported that lactulose excretions were significantly low in control subjects compared to diabetic patients. Similarly, a urinary excretion of L-rhamnose was significantly low in normal subjects compared to diabetic patients. However, subjects with type 1 diabetes (insulin-dependent diabetes) had significantly higher urinary lactulose excretion compared to type 2 diabetes (non-insulin-dependent diabetes), the urinary excretion of L-rhamnose and L/R ratio were not significantly higher in type 1 diabetic subjects [183]. In another short-term crossover clinical trial, the effects of lactulose supplementation in biscuits on day-time glucose, insulin, and amino acid concentrations were studied with 10 obese patients. All patients had normal or high-normal fasting blood glucose (average fasting blood glucose was 5.2 ± 0.6 mmol L^−1^), but only two subjects had normal glucose tolerance in response to oral glucose administration. Four subjects were classified as impaired glucose tolerance and four subjects had diabetes mellitus. All patients received three biscuits at breakfast, four biscuits during lunch and four biscuits during evening meal. The recipe of lactulose-fortified biscuit was 10 g of dietary fiber, 2 g of raw fiber and 8.2 g of lactulose. It was reported that average day-time glucose and insulin level were significantly decreased due to lactulose supplemental biscuit intake [184]. 

## 5. Conclusions, Remarks and Future Prospects

Prebiotics galacto-oligosaccharide, lactosucrose, tagatose, lactulose, lactitol, and bionic acid are produced through different enzymatic- and microbial-bioconversions of lactose. They have unique biological activities and the Federal Food and Drug Administration (FDA) has declared them ‘safe’. However, whereas lactose-based prebiotics are stable in the upper intestinal tract, they are converted to lactic acid and short chain fatty acids (acetic acid, propionic acid and butyric acid), and gases (carbon dioxide, methane, hydrogen) in presence of gut microbiota. Physicians frequently recommend consumption of lactose-based prebiotics with fruit juices and dairy products, and in some cases, with probiotics to individuals of all ages. When lactose-based prebiotics are consumed alone, their biological activities are expressed via interaction with already existing gut microbiota. Consumption of lactose-based prebiotics with probiotics offers some extra advantages due to the symbiotic activity. Results of several clinical investigations indicate that galacto-oligosaccharide can reduce the risks of osteoporosis and hyperlipidemia. Lactulose can reduce the risks of osteoporosis, hyperlipidemia, and hyperglycemia.

However, although lactose-based prebiotics are confirmed as safe, over consumption of them can cause osmotic diarrhea, dehydration, abdominal pain, and vomiting. Doses of galacto-oligosaccharide and lactulose are adjusted to 7.5 to 15 g day^−1^ for 7 to 21 days and 3 to 20 g day^−1^ for 14 to 28 days, respectively to ensure 2 to 4 bowel movements per day [236]. Nevertheless, probiotics are considered as ‘Generally Regarded As Safe’ (GRAS), in some cases, probiotics offer negative outcomes. In children with a short bowel syndrome, over production of toxic metabolites, such as D-lactate can be related with high consumption of probiotics along with lactose-based prebiotics or normal diet [237]. Predominance of *Bacillus subtilis* in infant formula is responsible for allergic and autoimmune diseases. In patients with short bowel syndrome, intake of *Lactobacillus* GG may create infection, such as endocarditis and bacteremia due to their translocation from the digestive tract to extra-intestinal sites. Furthermore, fungemia, due to contamination with *Saccharomyces* spp. in central catheters in patients who had jejunostomy, cancer, multiple comorbidities, and were immunocompromised, has been reported on several occasions [238,239]. However, whereas some *Bacillus* spp., such as *Bacillus subtilis*, *Bacillus coagulans*, *Bacillus cereus*, *Bacillus clausii*, and *Bacillus licheniformis* are used as a probiotic, food fortification, and food-grade biomolecule production, their applications as probiotics are an issue of debate from a safety point of view [240]. However, the optimum dose of each probiotic strain and their durability are unknown. Commercially available probiotic formulations generally have 10^6^–10^12^ colony forming units of probiotics day^−1^ [241]. Random consumption of unknown or non-recommended probiotics deplete the equilibrium of microbial community in the intestine and provide antibiotic resistance to unfavorable consortia in the gut due to antibiotic resistance plasmids transfer to commensal bacteria from probiotics *Lactobacillus* and Bifidobacteria. [242]. However, consumption of several or unknown probiotics may enhance nonspecific immune responses, and their effect on adaptive cellular and humoral immune responses are potentially significant [243]. Up to now, a substantial number of investigations have been performed with monoculture of lactic acid bacteria, such as *Lactobacillus*, Bifidobacteria [244], and commercial mixed culture VSL#3 [245]. Few studies have been published about other probiotics, such as *Enterococcus*, *Pediococcus*, *Leuconostoc*, *Lactococcus*, *Streptococcus*, [246] and other commercial lactic acid bacterial culture, such as Probio-Tec^®^, Culturelle^®^, Actimel^®^ (DanActive), Activia^®,^ and Yakult^®^ [245]. Information about several non-lactic acid probiotic bacteria, including *Bacillus*, *Clostridia*, *Propionibacteria*, and *Escherichia coli* Nissle 1917 is limited [246].

Although some clinical investigations have been performed with lactose-based prebiotics, mainly galacto-oligosaccharide and lactulose in this context, many more judicious investigations are required with human models to demonstrate their mechanisms, safety, efficiencies, and limitations. Furthermore, clinical investigations with other lactose-based prebiotics, such as lactitol and lactosucrose are needed to understand their effectiveness against osteoporosis, hyperlipidemia, and hyperglycemia. As biochemical activities of lactose-based prebiotics are expressed in a better way in the presence of probiotics, future challenges shall be to find out suitable strains, the introduction of the newer generation of probiotics, identify their metabolic pathways, synthesized metabolites, and their biochemical importance. Several clinical investigations have argued that, due to the wide range of microbial diversity in terms of activity and associate biochemical mechanisms among similar genus and even within species, it is not worth generalizing and comparing the potentialities of probiotics. Their application is dose-, age-, and situation-dependent. Therefore, a great emphasis is needed on the accumulation of knowledge about exact genus and specie of both lactic acid- and non-lactic- acid bacteria by high throughput sequencing and advanced bioinformatics. To understand their activities, more specialized in vitro and in vivo investigations are necessary. Furthermore, systematic and judicious investigations are a prerequisite to find out their optimum dose, mode of administration, and associated safety.

Direct disposal of whey in aquatic systems is forbidden due to presence of high concentration of lactose in whey. In the context of ‘Waste valorization’, production of different types of prebiotics from whey or de-proteinated whey via enzymatic-biotransformation as well as microbial fermentation processes may be a unique approach, rather than the direct disposal of whey into the aquatic system. In the cutting-age area in biotechnology, this approach can be a two-fold solution to the questions related to the biotechnological economy and recycling strategy. Furthermore, it is expected that this review will receive the attention of medical practitioners, food, and nutrition research communities.

## Figures and Tables

**Table 1:** Biochemical mechanisms for the synthesis of lactose-based prebiotics and biological outcomes due to symbiosis of lactose-based prebiotics and probiotics (self-developed, information were collected from Nath et al., 2016 [7]; Nath et al., 2017 [8]).

Lactose-Derived Prebiotics	Reaction Mechanisms	Biochemical Activities
Galacto-oligosaccharide	Transgalactosylation of lactose, galactose and glucose	Prevention of diarrhea, constipation, hyperlipidemia, and osteoporosis
Lactulose	Isomerization of lactose	Prevention of Crohn’s disease, ulcerative colitis, hepatic encephalopathy, constipation, hyperlipidemia, hyperglycemia, and osteoporosis
Lactitol	Reduction of lactose	Prevention of hepatic encephalopathy and constipation
Lactosucrose	Fructosyl transfer	Prevention of Crohn’s disease and ulcerative colitis
Lactobionic acid	Oxidation of lactose	Antioxidant and ultraviolet protector
Gluconic acid	Oxidation of glucose	Antioxidant and ultraviolet protector
